# Unilateral Nasal Obstruction during Later Growth Periods Affects Craniofacial Muscles in Rats

**DOI:** 10.3389/fphys.2016.00669

**Published:** 2017-01-10

**Authors:** Karin H. Uchima Koecklin, Maya Hiranuma, Chiho Kato, Yukiha Funaki, Taku Kataguchi, Tadachika Yabushita, Satoshi Kokai, Takashi Ono

**Affiliations:** ^1^Orthodontic Science, Department of Oral Health Sciences, Graduate School of Medical and Dental Sciences, Tokyo Medical and Dental UniversityTokyo, Japan; ^2^Faculty of Dentistry, Tokyo Medical and Dental UniversityTokyo, Japan

**Keywords:** digastric muscle, electric stimulation, genioglossus muscle, mouth breathing, oxygen saturation

## Abstract

Nasal obstruction can occur at different life stages. In early stages of life the respiratory system is still under development, maturing during the growth period. Previous studies have shown that nasal obstruction in neonatal rats alters craniofacial function. However, little is known about the effects of nasal obstruction that develops during later growth periods. The aim of this study was to investigate the effects of nasal obstruction during later periods of growth on the functional characteristics of the jaw-opening reflex (JOR) and tongue-protruding muscles. In total, 102 6-day-old male Wistar rats were randomized into either a control or experimental group (both *n* = 51). In order to determine the appropriate timing of nasal obstruction, the saturation of arterial oxygen (SpO_2_) was monitored at 8 days, and at 3, 5, 7, 9, and 11 weeks in the control group. Rats in the experimental group underwent unilateral nasal obstruction at the age of 5 weeks. The SpO_2_ was monitored at 7, 9, and 11 weeks in the experimental group. The electromyographic responses of JOR and the contractile properties of the tongue-protruding muscles were recorded at 7, 9, and 11 weeks. In the control group, SpO_2_ decreased until 5 weeks of age, and remained relatively stable until 11 weeks of age. The SpO_2_ was significantly lower in the experimental group than in the control. In the experimental group, JOR changes included a longer latency and smaller peak-to-peak amplitude, while changes in the contractile properties of the tongue-protruding muscles included larger twitch and tetanic forces, and a longer half-decay time. These results suggest that nasal obstruction during later growth periods may affect craniofacial function.

## Introduction

Mouth breathing is a concern in various fields of medical care because it has several health repercussions (Pacheco et al., 2015). Changes in the breathing pattern can occur owing to obstruction in the nasopharyngeal region associated with conditions such as tonsillar and adenoidal hyperplasia, hypertrophied turbinates, and rhinitis, increasing nasal resistance to air passage (Marcus, 2000; Juliano et al., 2009; Pacheco et al., 2015).

Nasal obstruction can occur at all life stages; airway obstruction by adenotonsillar hypertrophy is particularly common in children aged 4–14 years (Yadav et al., 2003). Moreover, the number of cases of respiratory complications related to allergic rhinitis and obstructive sleep apnea is increasing, especially during preadolescence and adolescence growth periods (Abreu et al., 2008; Juliano et al., 2009; Izu et al., 2010). The earlier the nasal obstruction develops, the earlier associated alterations can occur, not only in the respiratory system, but also in the whole body.

Nasal obstruction can lead to an imbalance in the musculature of the face, affecting craniofacial morphology and function (Sousa et al., 2005; Juliano et al., 2009; Pacheco et al., 2015). A normal respiratory pattern is associated with normal craniofacial structures and adequate interaction between mastication and swallowing (Yamada et al., 1997). Experimental studies have elucidated that nasal obstruction induces a significant reduction in the vertical development of the nasomaxillary complex and skull base along the longitudinal axis (Scarano et al., 1998). It also results in a significant reduction in the growth of the masseter and the anterior digastric (Dig) muscle (Gelhaye et al., 2006; Izu et al., 2010). With regard to masticatory function (Hsu and Yamaguchi, 2012; Ikenaga et al., 2013), previous studies have shown that oral breathing reduces masseter muscle activity (Ferla et al., 2008), and increases suprahyoid muscle activity and hypotonia of the lips and buccinator muscle (Valera et al., 2003). In our previous electrophysiological study, we suggested that unilateral nasal obstruction during early periods of growth reduces the response properties of the jaw-opening reflex (JOR; Funaki et al., 2014), increases the contraction force of the tongue-protruding muscles, and prolongs the duration of muscle contraction (Uchima Koecklin et al., 2015). The JOR plays an important role in the regulation of jaw movement during mastication (Lund, 1991). However, the effects of nasal obstruction at different life stages according to the maturity of breathing function are still not well understood.

The aim of this study was investigate the effects of unilateral nasal obstruction during later periods of growth, when the respiratory function has matured, and to evaluate JOR functional characteristics and contractile characteristics of the tongue protruding muscles. We focused on the saturation of arterial oxygen (SpO_2_) because this is directly influenced by respiratory rate and depth.

The hypothesis of the present study was that unilateral nasal obstruction at late stages of growth alters both the jaw-open reflex regulation of the masticatory muscles and the physiological contractile characteristics of the tongue-protruding muscles.

By understanding whether the changes in these different growth periods are similar or not, we may have more clues concerning the therapeutic approach in children and adolescents with alterations in the breathing pattern, and their different problems they have in the craniofacial complex and its function.

## Materials and methods

The experimental procedures described here were approved by the Animal Welfare Committee and performed in compliance with the Animal Care Standards of Tokyo Medical and Dental University (#0150160A and #0150161A).

### Recording of SpO_2_ in normal rats

To investigate the effects of nasal obstruction during growth on the respiratory function, we recorded SpO_2_ in 60 6-day-old Wistar albino rats. SpO_2_ was also recorded when the rats reached 8 days, and 3, 5, 7, 9, and 11 weeks of age (*n* = 10 per group per time point). We measured the SpO_2_ with a pulse oximeter (MouseOx; STARR Life Sciences Corp., Oakmont, PA, USA) placed on the neck (Carreras et al., 2011). We monitored the SpO_2_ alongside simultaneous observations of rat behavior in a chamber; rats were recorded for no longer than 2 h to avoid circadian effects. The size of the chamber and flow of fresh air were adequate for the rats (Han et al., 2002; Mortola, 2007). The SpO_2_ signal was digitally sampled at 1 Hz, stored, and analyzed. The mean SpO_2_ was obtained with a time constant of 10 s in inactive rats.

### Animal preparation for nasal obstruction

A total of 102 6-day-old male Wistar albino rats were weighed and then randomized into either a control (*n* = 51) or experimental group (*n* = 51) at 5 weeks of age. All rats were lightly anesthetized with 60 mg/kg of thiamylal sodium (Isozol®, Nichi-Iko Pharmaceutical Co., Toyama, Japan) administered intraperitoneally (i.p.). Rats in the experimental group received a left-sided nasal obstruction via the cauterization of the left external nostril (Scarano et al., 1998; Padzys et al., 2011). The tissue surrounding the left external nostril was burned by placing a surgical cauterizing instrument (Hakko Red, Hakko Corporation, Osaka, Japan) on the nostril, consequently occluding the orifice of the nostril without mechanical or chemical damage to the olfactory mucosa. After cauterization, the nostril was coated with 3% chlortetracycline (Aureomycin® Ointment; Pola Pharma, Tokyo, Japan) to prevent infection. The control group received a sham operation by cauterizing the skin ~1–2 mm above the left nostril.

The body weights of rats in both groups were monitored throughout the entire experimental period to assess their general health status. SpO_2_ was recorded in both groups when the rats were 7, 9, and 11 weeks of age (*n* = 10 per group per time point), without anesthesia. Following this, JOR was recorded from the Dig muscle in both groups at 7, 9, and 11 weeks of age (*n* = 10 per group per time point). The same rats were used in both procedures. In addition, the contractile characteristics of the tongue-protruding muscles were recorded at 7, 9, and 11 weeks of age (*n* = 7 per group per time point).

### Stimulation and recording of JOR

For electrophysiological recordings, all rats were lightly anesthetized with 60 mg/kg of i.p. thiamylal sodium (Isozol®, Nichi-Iko Pharmaceutical Co.). The depth of anesthesia was monitored by checking the pupil size, flexor and corneal reflexes, and heart rate. When a firm pinch applied to the tail increased respiratory and heart rate, a supplemental injection of 5 mg/kg of i.p. thiamylal sodium was administered. The rectal temperature was maintained at 37°C with a heating pad. A short-acting local anesthetic (10 μL of 2% lidocaine; AstraZeneca Canada Inc., Mississauga, ON, Canada) was infiltrated subcutaneously prior to incision of the facial skin.

**Figure 1:**
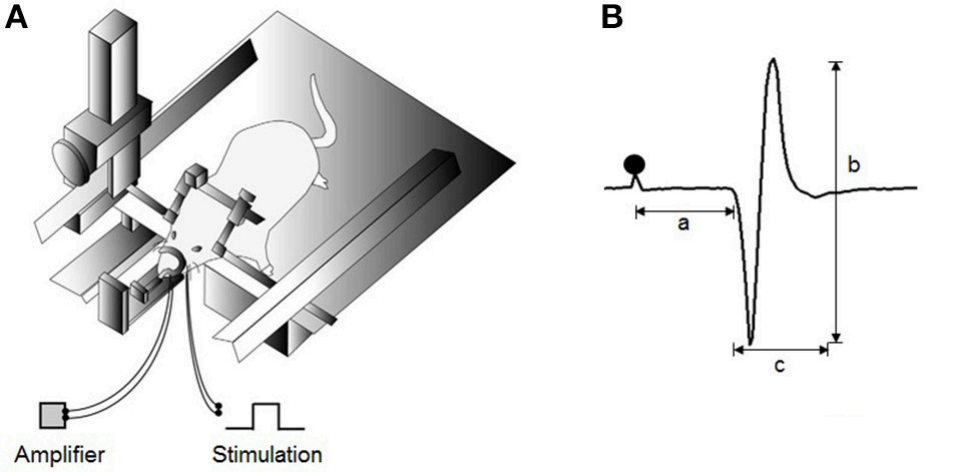
**Experimental design for jaw-opening reflex (JOR) measurements. (A)** Schematic drawing of the experimental setting for evoking JOR. The heads of anesthetized rats are fixed to a stereotaxic frame. JOR is evoked by electrical stimulation of the bilateral inferior alveolar nerve (IAN), and electromyographic (EMG) activity is recorded from the anterior belly of the bilateral digastric (Dig) muscles. **(B)** Typical example of evoked JOR. Filled circles indicate stimulation artifacts. a, latency; b, peak-to-peak amplitude; c, duration. Dot depicts the timing of stimulation.

To stimulate the left inferior alveolar nerve (IAN), a pair of stainless-steel wire electrodes (0.1 mm in diameter, 0.5 mm tip exposure) was inserted into the left mandibular canal through the mental foramen at depths of 1 and 3 mm. The bipolar electrode was kept in place by fixing it onto the adjacent mandibular bone with a light-curing dental resin. JOR expression was recorded from the left Dig muscle using a pair of stainless-steel wire electrodes with 1-mm exposed tips implanted along the direction of the muscle fibers. The interpolar distance was set at 3 mm between these recording electrodes. To ensure consistent reproduction of the distance, a two-keyhole index, with the keyholes 3 mm apart, composed of dental acrylic (UNIFAST II; GC Corporation, Tokyo, Japan) was used to lead the paired electrode-inserted needles. The animals were then transferred to a stereotaxic apparatus (models SN-2 and SM-15M; Narishige Scientific Instruments, Tokyo, Japan) with their bodies in prone position (Figure 1A). Before stimulation, the Dig electromyographic (EMG) baseline activity was captured for 1 min.

The IAN was stimulated electrically (single pulse, 0.2 ms in duration) once every 5 s to evoke JOR. The stimulus intensity was gradually increased at 1 mA increments, and the lowest intensity that constantly elicited a Dig EMG response was determined as the JOR threshold (T). To attain comparable responses, the test stimulation current was adjusted to 1.5-times that of that of the threshold (1.5T; Funaki et al., 2014). An intensity of 2T or lower was considered non-nociceptive stimulation.

Electromyography (EMG) activity was amplified by a 100-Hz to 3-kHz bandwidth differential amplifier (DAM-80; WPI, Sarasota, FL, USA). All signals were fed into a computer by means of a CED 1401 interface (5000/s sampling rate) and were later analyzed offline with Spike2 software for Windows, version 4.02 (Cambridge Electronic Design, Cambridge, UK). After recording, the animals were euthanized via an i.p. overdose of thiamylal sodium. The Dig muscle was then dissected to confirm the location of the placed electrode.

JOR was assessed according to three response properties: latency, duration, and peak-to-peak amplitude (Figure 1B). The mean values of these parameters were averaged from the reflex responses after 30 consecutive stimuli in each rat. EMG activities were full-wave rectified and smoothed at a time constant of 20 ms. The latency and duration were indicated as time intervals in ms. The latency was defined as duration between the stimulus and the first point at which Dig EMG activity exceeded 2 standard deviations (SD) of the baseline activity (i.e., onset), whereas the duration was defined as the duration between the onset and the point at which the response dropped below 2 SD of the baseline activity (i.e., offset). The peak-to-peak amplitude in mV was calculated between the onset and offset in each single sweep.

### Recording of the tongue-protruding muscles contractile characteristics

All rats were anesthetized with 70 mg/kg i.p. ketamine (Ketalar, Daiichi Sankyo, Tokyo, Japan) and 7 mg/kg xylazine (Seractal, Bayer, Tokyo, Japan). The depth of anesthesia was monitored by checking the pupil size, flexor and corneal reflexes, and heart rate. A short-acting local anesthetic (10 μL of 2% lidocaine; AstraZeneca Canada Inc.) was infiltrated subcutaneously prior to incision of the facial skin. The hypoglossal nerves were then exposed on both sides by a ventral approach. A middle incision at the anterior neck was performed, and bifurcation of the hypoglossal nerve was identified deep at the intersection of the lateral edge of Dig muscle's anterior belly and the edge of the mylohyoid muscle. The medial branch of the hypoglossal nerve innervates the genioglossus and intrinsic muscles of the tongue, and the lateral branch innervates the retraction muscles of the tongue. Therefore, a 2- to 5-mm section of the lateral branch of the hypoglossal nerve was removed bilaterally to allow stimulation of the tongue-protruding muscles.

**Figure 2:**
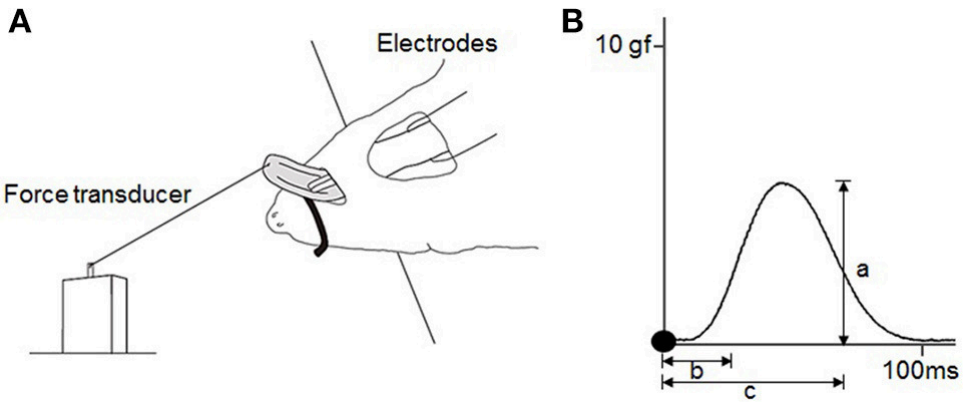
**Experimental design for measurements of the contractile properties of the tongue-protruding muscles. (A)** Hypoglossal nerves are exposed bilaterally after a mid-sagittal incision at the anterior neck. Bifurcation of the hypoglossal nerve into the medial and lateral branches are found deep at the intersection of the lateral edge of the anterior belly of digastric muscle and the edge of the myohyoid muscle. The lateral branch of the hypoglossal nerve is transected, and only the medial branch of the hypoglossal nerve is stimulated. The rat is held in a supine position and the tip of the tongue is connected to the force transducer by placement of a suture. **(B)** Typical twitch force for measurement of a = maximal twitch force, b = CT, c = HDT. Filled circles indicate the onset of stimulation. To measure the magnitude of the tetanic forces at 60 and 80 Hz, and in the FI, the same procedure as that for the maximal twitch force is adopted (a). The FI percentage is the division of the first force by the last force after 2 min of stimulation.

To measure the contractile properties of the tongue-protruding muscles, a 20-cm-long 3–0 black silk suture (Matsuda Sutures, Tokyo, Japan) was attached to the tip of the tongue and connected the end of the suture to an isometric force transducer (MLT0420; ADInstruments, Dunedin, New Zealand). The rat was placed onto a stage with the body and head fixed, and the tongue extended at a preloaded force of 3 g (Figure 2A), as described previously (Uchima Koecklin et al., 2015).

The measured parameters of muscle contraction included twitch force, contraction time (CT), half-decay time (HDT), tetanic force at 60 Hz, tetanic force at 80 Hz, and fatigue index (FI). Peak twitch force is the maximum tension generated from single supramaximal stimulation (1.5-times the maximal stimulation) of the specific motor nerve. Tetanic force is generated by repeated stimulations applied before the occurrence of complete relaxation and is a fused force signal. Both forces were measured from the onset of the stimulation to the peak of the curve force. The FI represents the reduction of tetanic force over 2 min of continuous stimulation, calculated by the first force divided by the last force after 2 min of stimulation. Temporal variables were measured from the twitch force signal. CT is the duration between the onset of the stimulation and the point of 50% peak force. HDT is the duration between the onset of stimulation and the point of 50% decay from peak force (Figure 2B).

We applied a bilateral electrical stimulation to the medial branches of the hypoglossal nerves by 0.1-ms rectangular pulses at a supramaximal current (generally ~500 μA), as described in previous studies (Uchima Koecklin et al., 2015). Electrical stimulation was supplied by an electric stimulator (SEN-7203, Nihon Kohden, Tokyo, Japan) with two tungsten electrodes (0.010-inch-length, 250-μm-diameter, epoxylite insulation, 9 MΩ of impedance measured at 1000 Hz; FHC, Bowdoin, ME, USA). Both electrodes were placed simultaneously, and the tip of the electrode was inserted into the medial branch proximal to the bifurcation of the hypoglossal nerve, with one electrode on each side.

Supramaximal stimulation was considered as 1.5-times that of the current level required to obtain maximal twitch force at the supramaximal magnitude. Stimuli were delivered at 1 Hz for twitch force while stimuli for tetanic force were delivered at 60 and 80 Hz for 200-ms trains. FI was recorded with a 2-min stimulation of a 100-Hz train for 500 ms. All signals were also analyzed with Spike2 software for Windows (Cambridge Electronic Design).

### Statistical analysis

All data were expressed as mean ± SD. An unpaired *t*-test was used for statistical comparison of body weight between the two groups. Repeated measures multivariate analysis of variance was conducted for the intergroup and intragroup comparisons. Simple main effects analysis using the Sidak adjustment was applied for multiple comparisons. Statistical analysis was performed using SPSS for Windows software, version 13.0J (SPSS Inc., Chicago, IL, USA); statistical significance was established at *P* < 0.05.

## Results

### Body weight

Mean body weight in the control and experimental groups increased continuously throughout the experimental period, and there were no significant differences between rats of the same age in the two groups. Weight is shown in Table 1.

**Table 1:** **Body weight**.

**Control**						**Experiment**					
**7w**		**9w**		**11w**		**7w**		**9w**		**11w**	
Mean	SD	Mean	SD	Mean	SD	Mean	SD	Mean	SD	Mean	SD
245.0	±24.3	314.2	±24.7	340.6	±6.2	231.0	±16.8	310.4	±18.1	340.2	±13.0

### SpO_2_

**Figure 3:**
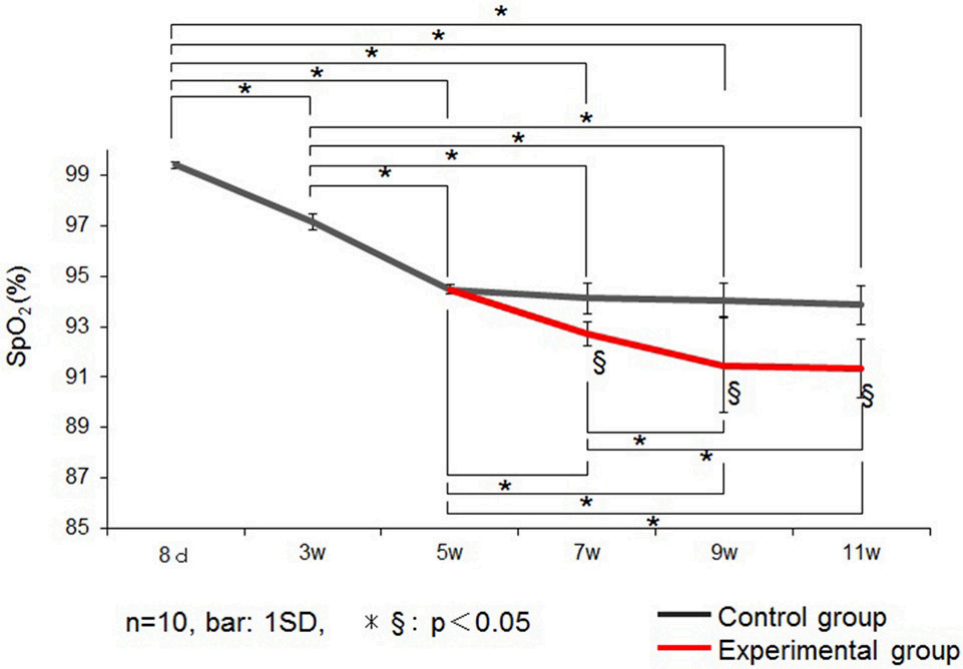
**Changes in the saturation of arterial oxygen (SpO_2_) (%) in the control and experimental groups**. Data are expressed as mean ± standard deviation. ^*^ and ^§^*P* < 0.05.

Changes in SpO_2_ is represented in Figure 3. In control group rats, the SpO_2_ peaked at 8 days, decreased until 5 weeks, and remained stable thereafter until 11 weeks of age. Therefore, in this experiment, we performed the nasal obstruction at 5 weeks of age.

SpO_2_ was significantly lower in the experimental group than in the control group at 7, 9, and 11 weeks of age.

### JOR

**Figure 4:**
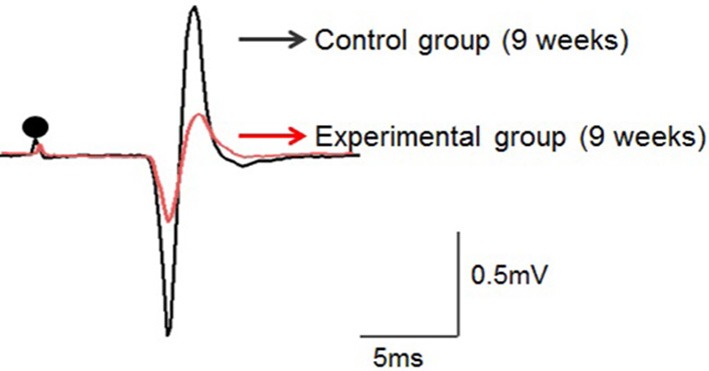
**Typical examples of the jaw-opening reflex (JOR) responses of rats in the two groups at 9 weeks of age**. The stimulus intensity is adjusted to 1.5-times the threshold (1.5T) in each rat. Dots depict the timing of stimulation. Superimposition of the JOR, black line indicates the control group, and red line the experimental group.

Low-intensity electrical stimulation of the left IAN elicited the JOR response in the left Dig muscle in all rats. Typical examples in both groups recorded at 9 weeks of age are shown in Figure 4.

**Figure 5:**
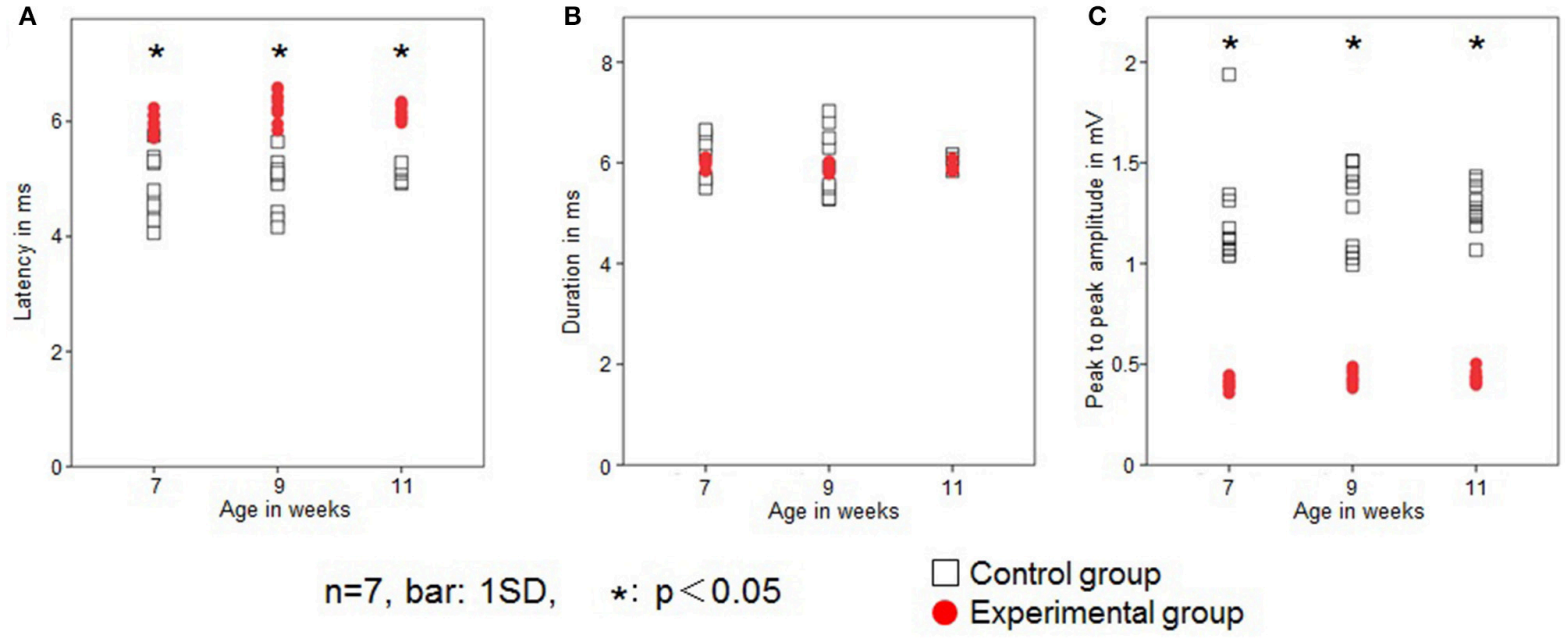
**Changes in the jaw-opening reflex (JOR)**. Latency (ms; **A**), duration (ms; **B**), and peak-to-peak amplitude (mV; **C**) in the two groups. Open square, control group; red dot, experimental group. Data are expressed as mean ± standard deviation. ^*^P < 0.05.

In the control group, JOR latency was 4.8 ± 0.5, 4.9 ± 0.4, and 5.1 ± 0.1 ms at 7, 9, and 11 weeks of age, respectively. In the experimental group, latency was 6.0 ± 0.2, 6.3 ± 0.2, and 6.2 ± 0.1 at 7, 9, and 11 weeks of age, respectively. The latency was significantly longer in the experimental group than in the control group at each age recording (Figure 5A). Intragroup comparison between 7, 9, and 11 weeks of age revealed no significant difference in JOR latency in either the control or experimental group.

JOR duration in the control group was 6.1 ± 0.4, 6.0 ± 0.1, and 6.1 ± 0.1 ms at 7, 9, and 11 weeks of age, respectively, while in the experimental group it was 6.0 ± 0.1, 5.9 ± 0.1, and 5.9 ± 0.1 ms, respectively. The JOR duration was not significantly different between the two groups (Figure 5B). Intragroup comparison among 7, 9, and 11 weeks of age revealed no significant differences in either the control or experimental group.

In the control group, JOR peak-to-peak amplitude was 1.2 ± 0.3, 1.3 ± 0.2, and 1.3 ± 0.1 mV at 7, 9, and 11 weeks of age, respectively, while in the experimental group, it was 0.4 ± 0.03, 0.4 ± 0.03, and 0.4 ± 0.03 ms, respectively. The peak-to-peak amplitude was significantly smaller in the experimental group than in the control group at each recording age (Figure 5C). Intragroup comparison between 7, 9, and 11 weeks of age revealed no significant differences in peak-to-peak amplitude in either the control or experimental group.

### Contractile properties of the tongue-protruding muscles

**Figure 6:**
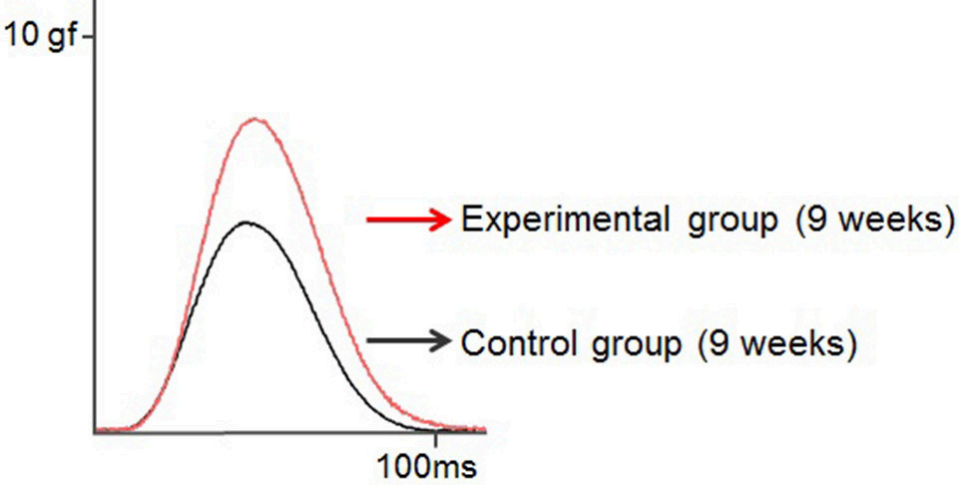
**Typical examples of the twitch force of the tongue-protruding muscles in the two groups at 9 weeks of age**. The supramaximal stimulation is adjusted to 1.5-times the current level required to obtain the maximal twitch force. Superimposition of the twitch force curves, black line indicates the control group, and red line the experimental group.

**Figure 7:**
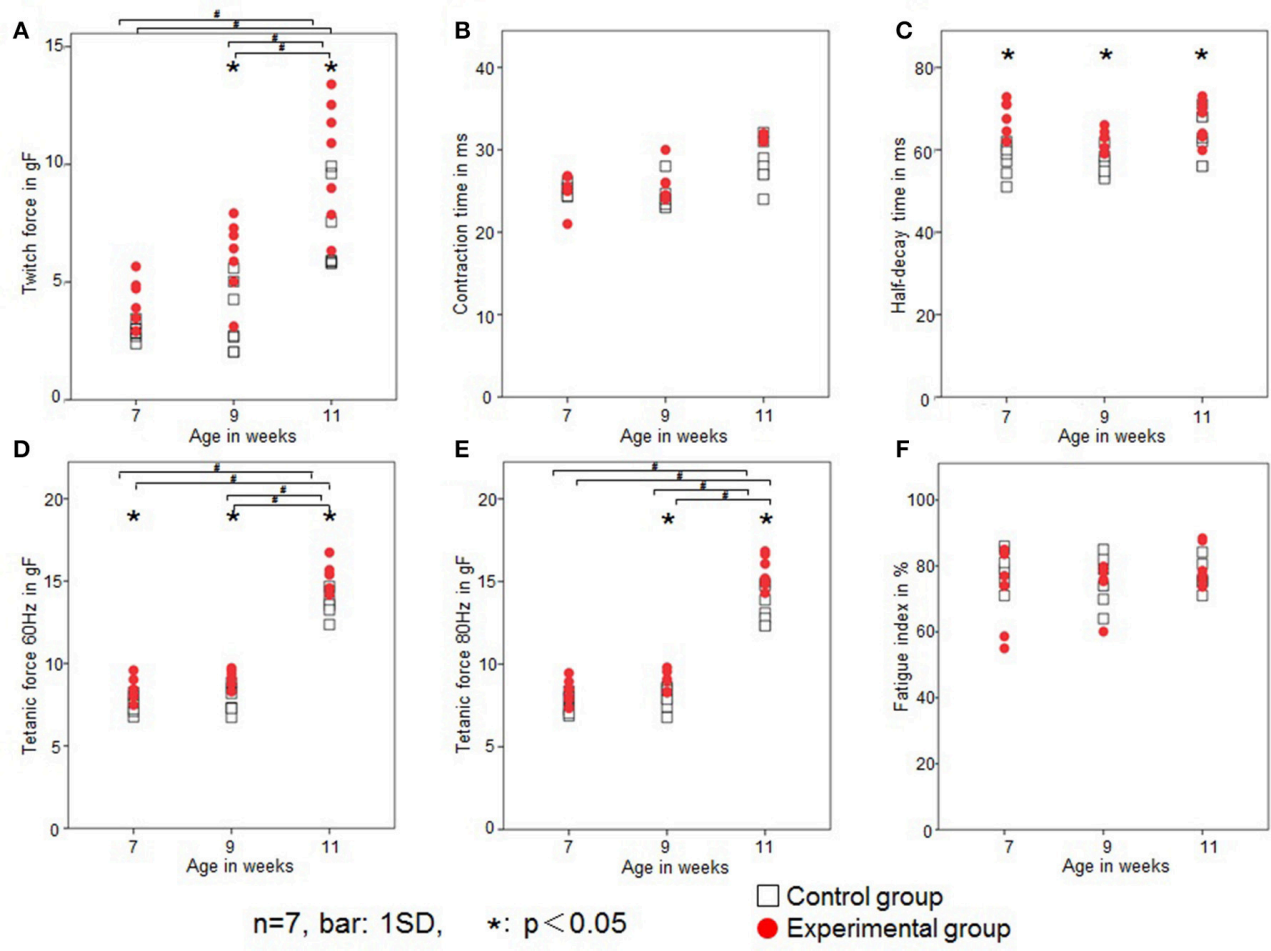
**Changes in the contractile properties of the tongue-protruding muscles**. Twitch force (gf; **A**), CT (ms; **B**), HDT (ms; **C**), tetanic force at 60 Hz (gf; **D**), tetanic force at 80 Hz (gf; **E**), and FI (%; **F**) in the two groups. Open square, control group; red dot, experimental group. Data are expressed as mean ± standard deviation. ^*^P < 0.05.

Rat twitch force signals recorded in both groups are shown in Figure 6. In the control group, the maximal twitch force was 2.9 ± 0.3, 3.5 ± 1.5, and 7.2 ± 1.9 gf at 7, 9, and 11 weeks of age, respectively, while in the experimental group, the maximal twitch force was 4.1 ± 1.1, 6.1± 1.6, and 10.3 ± 2.6 gf, respectively. The twitch contraction force significantly increased at 9 and 11 weeks of age in the experimental group (Figure 7A). Intragroup comparisons revealed significant differences between the 7- and 11-week-old and between the 9- and 11-week-old rats of the control group, and between the 7- and 11-week-old and between the 9- and 11-week-old rats of the experimental group.

In the control group, the CT was 25.1 ± 0.7, 25.2 ± 1.5, and 28.3 ± 2.7 ms at 7, 9, and 11 weeks of age, respectively, while in the experimental group, the CT was 26.6 ± 4.0, 26.1 ± 1.9, and 29.2 ± 2.5 ms, respectively. There were no significant differences in CT between the control or experimental group (Figure 7B). Intragroup comparisons revealed no significant differences in CT in either the control or experimental group.

In the control group, the HDT was 57.8 ± 3.9, 57.9 ± 3.6, and 62.7 ± 5.6 ms at 7, 9, and 11 weeks of age, respectively, while in the experimental group, it was 68.5 ± 4.0, 63.5 ± 2.5, and 67.4 ± 4.6 ms, respectively. The HDT significantly increased in the experimental group at all ages compared with the control group (Figure 7C). Intragroup comparisons among 7-, 9-, and 11-week-old animals revealed no significant differences in HDT in either the control or experimental group.

In the control group, the tetanic force at 60 Hz was 7.6 ± 0.5, 7.7 ± 0.8, and 13.6 ± 0.7gf at 7, 9, and 11 weeks of age, respectively, while in the experimental group, the tetanic force at 60 Hz was 8.4 ± 0.7, 9.2 ± 0.5, and 15.2 ± 0.9 gf, respectively. In the control group, the tetanic force at 80 Hz was 7.5 ± 0.6, 7.9 ± 0.7, and 13.4 ± 1.1 gf at 7, 9, and 11 weeks of age, respectively, while in the experimental group, it was 8.4 ± 0.7, 8.9 ± 0.6, and 15.5 ± 1.0 gf, respectively.

Tetanic forces at 60 Hz were significantly higher at all ages (Figure 7D), and at 80 Hz were significantly higher in the experimental group at 9 and 11 weeks of age (Figure 7E). Intragroup comparisons revealed significant differences for both 60 and 80 Hz tetanic forces, between the 7- and 11-week-old and between the 9- and 11-week-old rats of the control group, and between the 7- and 11-week-old and between the 9- and 11-week-old rats of the experimental group.

In the control group, the FI was 79 ± 5, 75 ± 7, and 77 ± 4% at 7, 9, and 11 weeks of age, respectively, while in the experimental group it was 72 ± 12, 75 ± 7, and 80 ± 6%, respectively. There was no significant difference in FI between the control and experimental group (Figure 7F). Intragroup comparisons revealed no significant differences in FI in either the control or experimental group.

## Discussion

In this study, we investigated the effects of nasal obstruction during later periods of growth on the respiratory function, and functional characteristics of JOR and tongue-protruding muscles, in Wistar albino rats. We show that nasal obstruction during later periods of growth has the potential to affect the craniofacial function.

### Unilateral nasal obstruction in animal models

In previous studies, it has been reported that unilateral nasal obstruction before maturation of the respiratory function is well tolerated and affects the craniofacial complex (Otani-Saito et al., 2001), such as JOR (Funaki et al., 2014) and the contractile properties of the tongue-protruding muscles (Uchima Koecklin et al., 2015). Unilateral obstruction also induces atrophy of the ipsilateral olfactory bulb, associated with olfactory deprivation in rats (Meisami, 1976; Baekey et al., 2009). Also, in the present study we found that unilateral nasal obstruction did not cause any alteration in the body weight.

Mammalians are born with immature lungs. In rats, lung structural changes, such as alveolar development and septal reconstruction, are considered complete by 3 weeks of age (Burri, 2006; Bolle et al., 2008); and between the 2 and 5 weeks of age, the capillary growth plays an important role in the optimization of gas exchange (Burri, 1974, 2006; Yokoyama, 1983; Tschanz et al., 2003; Bolle et al., 2008). At this stage, capillary growth by intussusception also plays an important role in further optimization of gas exchange. In our study, SpO_2_ in the control group was stable by 5 weeks of age, when the rats are in a later period of growth. In addition, by this stage rats are during the growth spurt, so the craniofacial complex could be affected by any alteration in the normal physiology. Therefore, this study investigated the effects of nasal obstruction from 5 weeks of age, on the JOR functional characteristics and the contractile characteristics of the tongue-protruding muscles. We also found that SpO_2_ levels decreased after unilateral nasal obstruction in rats. It is possible that this low level of oxygen could be related to nasal obstruction, due to a reduction in the airflow, as seen in humans with nasal obstruction (Yadav et al., 2003; Niaki et al., 2010).

### Changes in JOR and contractile characteristics of the tongue-protruding muscles after nasal obstruction

In our previous studies, we performed unilateral nasal obstruction in neonatal rat pups at 8 days of age, before the stabilization of SpO_2_ levels (Funaki et al., 2014; Uchima Koecklin et al., 2015). The maturation of JOR was altered (Funaki et al., 2014) and the force of the tongue-protruding muscles increased (Uchima Koecklin et al., 2015). The present study also found similar results as discussed below.

At 5 weeks of age, the maturation of JOR and functional changes in the lungs are completed in rats (Funaki et al., 2014). Also, the response properties of rat periodontal mechanoreceptors and temporomandibular joint mechanoreceptors attain maturation by the age of 5 weeks (Nasution et al., 2002; Ishida et al., 2009). Since each receptor of the trigeminal nerve endings complete maturation at 5 weeks of age, nasal obstruction at this point is expected to have a significant effect on the stomathognatic system.

The JOR permits the rhythm regulation of the jaw movement. Previous reports in rats have suggested that nasal obstruction is associated with reduced growth of the masseter muscle and anterior digastric muscle (Gelhaye et al., 2006). The complex activities performed during the orofacial behaviors are possible by the activity of the different muscles, and the masticatory muscles coordination is regulated by several reflexes, such as the jaw-opening reflex, and the jaw-closing reflex (Funaki et al., 2014) Based on this, we decided to investigate the JOR, which helps in the different activities performed in the craniofacial complex, especially in mastication. The assessment of the JOR can be done via the digastric muscle, as this is a participant in the opening movement of the mandible. Alteration in the JOR could affect the response action of the masticatory muscles, that act in the closing of the mandible.

As rats grow, the JOR latency shortens (Thexton and Griffiths, 1979). However, the reduction in latency stops just before weaning, usually at ~3 weeks of age (Thexton et al., 1995). Latency is associated with a conduction velocity of nerve fibers; it prolongs myelination and increases axon diameter (Thexton et al., 1995; Lizarraga et al., 2007). Moreover, alteration of the peripheral sensory regulation system may induce a delay in conduction velocity, affecting the periodontal mechanoreceptors (Seki et al., 2002). In this study, nasal obstruction increased latency at all ages, in accordance with our previous experiment (Funaki et al., 2014). Therefore, we assume that nasal obstruction reduces the conduction velocity, resulting in delayed maturation of reflex regulation.

JOR duration is related to the number of muscle fibers contributing to the motor unit (Saboisky et al., 2012). In general, the number of muscle fibers decreases when muscle atrophy occurs (Timson and Dudenhoeffer, 1990). In this study and our previous study (Funaki et al., 2014), there was no significant difference in JOR duration between the control and experimental groups. Therefore, we speculate that nasal obstruction did not induce muscle atrophy. Peak-to-peak amplitude is calculated as the voltage difference of the motor unit potential, determined by the diameter and number of muscle fibers closest to the electrode (Saboisky et al., 2012). The peak-to-peak amplitude decreases when the periodontal ligament becomes narrower, and the periodontal mechanoreceptors degenerate (Santilakanawong et al., 2003). Nasal obstruction may weaken the occlusal force, triggering a reduction in periodontal mechanoreceptor stimulation. In the present study, peak-to-peak amplitude was significantly smaller after nasal obstruction at all ages, as reported in our previous study (Funaki et al., 2014).

Previous studies have shown that the genioglossus muscle, the main tongue protruder, increased its electromyographic activity after nasal obstruction (Miller, 1978). The tongue complex musculature permit the rapid and accurate movements of the tongue that are necessary for the different orofacial behaviors, such as respiration, deglutition, mastication, and phonation (Uchima Koecklin et al., 2015); especially the tongue protruding muscles, that play an important role in the maintenance of the upper airway diameter. The study of the contractile properties of the tongue-protruding muscles may help in the understanding of parafunctions related to nasal obstruction and mouth breathing, since the movements of the tongue are also governed by their muscle contractile properties (Sawczuk and Mosier, 2001).

Regarding the contractile properties of the tongue-protruding muscles, both the twitch and tetanic forces increased after nasal obstruction, as in our previous report (Uchima Koecklin et al., 2015). The twitch force is the smallest contractile response of the muscle fiber to stimulation, and is related to the elongation capacity of the muscle fiber (MacIntosh et al., 2006). In this study, the twitch force was elevated in 9- and 11-week-old rats after nasal obstruction. The tetanic force is the response of the muscle fiber to multiple consecutive stimulations, representing the overall capacity of muscle force (MacIntosh et al., 2006). In the present study, the rats with nasal obstruction showed increased tetanic forces at 60 Hz at all ages. However, only 9- and 11-week-old rats showed increased tetanic force at 80 Hz after nasal obstruction. The fusion of the tetanic wave force was between 60 and 80 Hz in all rats.

The increase in muscle force is related to increments in the cross-sectional area of the muscle fibers and fiber type (MacIntosh et al., 2006; Hellyer et al., 2012; Uchima Koecklin et al., 2015). Our results showed that the complete adaptation of the muscle, reflected in the increase in both the twitch and tetanic forces, occurred at 4 weeks after nasal obstruction, in the 9-week-old rats, assuming that this is the necessary time for rats' muscles to adapt. This is in accordance with a previous investigation (Holy and Zérath, 2000) where changes in muscle fibers were reported within 4 weeks after the beginning of training of the soleus muscle in growing female rats. Moreover, rats that underwent 10 Hz of chronic stimulation in muscles exhibited changes in muscle fibers 28 days later (MacIntosh et al., 2006). These changes in muscle fibers may be related to changes in the mitochondrial volume, as previously observed after 28 days of chronic stimulation in rabbit tibialis anterior muscles (MacIntosh et al., 2006).

CT is related to the velocity response of the muscle to stimulation. We found that CT was not altered after nasal obstruction, as reported in our previous study (Uchima Koecklin et al., 2015). HDT is associated with the maintenance of the steady force, the longer the duration, the longer the contraction force is maintained. In the present study, HDT increased at all ages after nasal obstruction, also in accordance with our previous study (Uchima Koecklin et al., 2015). The FI refers to the fatigability of the muscle, related to the muscle fiber type (Uchima Koecklin et al., 2015). The FI was unaffected by nasal obstruction suggesting that the tongue-protruding muscles had more fatigue resistant fibers. Our findings suggest that the contractile characteristics of the tongue-protruding muscles are altered with nasal obstruction.

### Effects of nasal obstruction in the craniofacial complex

In mammals, tongue movement is coordinated with the movements of the jaw to perform different orofacial functions (Lowe, 1980; Aeba et al., 2002). In mastication, the jaw-opener and tongue-protruding muscles are active during the jaw-opening phase, while the jaw-closer and tongue retractor muscles work in the jaw-closing phase (Aeba et al., 2002). In addition, the position of the tongue is controlled reflexively by the position of the jaw (Ishiwata et al., 1997, 2000). Therefore, JOR alterations may be related to changes in the tongue-protruding muscles. A previous study showed that the periodontal mechanoreceptors after tooth contact during chewing play a role in the short bursts of activity of both the Dig and genioglossus muscles (Kakizaki et al., 2002). During nasal obstruction masticatory activity is reduced (Hsu and Yamaguchi, 2012; Ikenaga et al., 2013), altering the periodontal mechanoreceptors, this may also be related to the changes in both the JOR and the tongue-protruding muscles.

JOR presented major changes after nasal obstruction from 7 weeks of age, while the contractile characteristics presented major changes from 9 weeks of age, suggesting that the complete adaptation of the JOR occurs before the complete adaptation of the contraction properties of the tongue-protruding muscles. In the stomathognathic system, the trigemino-hypoglossus reflex is related to the tongue muscles (Aeba et al., 2002), as stimulation in the trigeminal nerve can evoke excitatory and inhibitory responses in the hypoglossal nerve. In the present study, the faster alteration of JOR after nasal obstruction may suggest an important role of JOR in the alteration of tongue-protruding muscle activity.

The stomathognatic system has several functions including mastication, swallowing, phonation, and respiration; both JOR and tongue-protruding muscles play important roles in these functions (Kakizaki et al., 2002; Bailey and Fregosi, 2004; Funaki et al., 2014). In addition, changes in craniofacial morphology are correlated with bite force variation (Raadsheer et al., 1999), and have potential repercussions for masticatory function.

Impaired nasal breathing could lead to mouth breathing in humans; however, only 4.3% of them are fully mouth breathers, while most present a combination of nasal and oral breathing (Ung et al., 1990). Patients with mouth breathing and nasal obstruction during childhood have shown alterations in normal morphology (Song and Pae, 2001; Matter et al., 2004; Proffit et al., 2007; Al Ali et al., 2015) and muscular function (Gross, 1974; Song and Pae, 2001; Funaki et al., 2014) in the craniofacial complex, leading to anomalies in this area. Possible changes in the craniofacial muscles performance could be related to the imbalance of these muscles, favoring for the alteration in the morphology seen in patients with nasal obstruction and mouth breathing.

In other mammals, such as monkeys and rats, nasal obstruction also leads to anomalies in the craniofacial complex (Gross, 1974; Yamada et al., 1997; Scarano et al., 1998). These studies suggest that nasal obstruction affect the craniofacial musculature, possibly to help improve the inspiration of air by widening the upper airway, possibly by moving the mandible downwards and the tongue forward, as explained in the alteration of the JOR, which would maintain the mouth open, and the increase in the tongue-protruding force and longer contraction maintenance, which would lead the tongue to a forward position.

For these reasons, we propose that nasal obstruction affects both the JOR response properties and the maturation of the reflex regulation, and the contractile properties of the tongue-protruding muscles, regardless of the growth stage at which the nasal obstruction initiates. These affections may lead to changes in the normal growth and development of the craniofacial complex, as well as changes in the normal respiratory and orofacial function.

## Conclusions

Unilateral nasal obstruction during later growth periods in rats induced changes in SpO_2_, and the modulation of JOR response characteristics and contractile characteristics of the tongue protruding muscles. Based on the important role of the tongue and masticatory muscles performance on the different functions such as mastication, deglutition, phonation, and respiration, these findings suggest that unilateral nasal obstruction occurring during the later growth period may greatly affect the craniofacial function in rats. Therefore, nasal obstruction should be taken into consideration to avoid any repercussions in the physiological functions, as well as any effects in the normal growth and development of the craniofacial complex.

## Author contributions

All authors contributed to the conception and design of research; KU, MH, CK, TK, performed experiments; all authors analyzed data; all authors interpreted results of experiments; KU and MH prepared figures; KU, MH, and SK drafted manuscript; KU, CK, SK, and TO edited and revised manuscript; all authors approved the final version of manuscript.

## Funding

This study was financially supported in part by Grants-in-Aid for Scientific Research (24593081, 15K20581-0) from the Japanese Ministry of Education, Culture, Sports, Science, and Technology.

### Conflict of interest statement

The authors declare that the research was conducted in the absence of any commercial or financial relationships that could be construed as a potential conflict of interest.
